# Distal radius fractures and distal ulna fractures among adults in a southern China county during the 11-year-period 2010 to 2020

**DOI:** 10.1097/MD.0000000000040109

**Published:** 2024-10-11

**Authors:** Zhe-Kang Huang, Wu Zeng, Jun Li, Jun-Feng Zhu

**Affiliations:** a Suichang County People’s Hospital, Lishui, Zhejiang, China.

**Keywords:** adults, AO classification, distal radius fractures, distal ulnar fractures, Q-type, ulnar styloid fractures

## Abstract

Most distal radius fractures are associated with distal ulnar fractures. However, there is still a lack of consensus on the incidence of different types of distal ulnar fractures among adults in China. Therefore, we analyzed the incidence of distal radial fractures with and without associated distal ulnar fractures among adults in a southern China county from 2010 to 2020. Registry data of 2333 patients (2351 sides) with a distal radius fracture from 2010 to 2020 underwent evaluation, encompassing parameters such as age, sex, distal radius fracture classification, fracture side, and distal ulnar fracture classification. Distal radial fractures were classified according to the AO/OTA classification. Distal ulnar fractures were examined using the Q-modifier classification. 1719 females (73.68%) and 614 males (26.32%) were included in the study. Compared to men, the incidence of distal radial fractures accompanying distal ulnar fractures in women was approximately 2.8 times higher. Additionally, 49.81% of distal radius fractures were associated with fractures of the distal ulna, while 46.44% were associated with fractures of the ulnar styloid. The most common fracture type was that of the ulnar styloid Q1 (93.73%). The mean age of female patients was 61.71 ± 12.13, while male patients had an average age of 50.63 ± 14.86. The Q1 type was the most common type of distal ulnar fracture. We also found that more females (age range: 50 years or older) had type C distal radius fractures compared to males. However, type B fractures were observed more frequently in males than in females (range: 18–49 years). Osteoporosis was believed to be the main cause of fractures in women aged >50 years old. Moreover, the peak incidence of radius fractures in males was lower than in females in different age groups.

## 1. Introduction

Distal radial fractures are commonly associated with distal ulnar fractures. The association between distal radial and distal ulnar fractures varies, with a higher incidence in elderly patients with osteoporosis.^[1]^ Isolated distal ulnar fractures are relatively rare; however, ulnar styloid fractures commonly occur in distal radial fractures.^[2]^ For instance, the findings by Moloney et al^[3]^ demonstrated an incidence of 74/100,000 person-years in adults living in Östergötland, Sweden, from 2010 to 2012. Fractures of the ulnar styloid were the most common (79% Q1), followed by fractures of the ulnar neck (11% Q2). In a different study, Herzberg and Castel concluded that 9% of patients with distal radial fractures also had distal ulnar fractures, excluding styloid fractures. They defined the distal ulna as the ulnar head and neck without further specification or the distal third of the ulna, as in the present study. Specifically, 5.9% of cases involved a fracture of the ulnar neck, 1.6% exhibited fractures of both the head and neck, and 1.4% manifested a fracture of the ulnar head.^[4]^

Distal radius and ulnar fractures are common in children, teenagers, and adults.^[5]^ However, most of studies have been conducted on this subject in China that discuss various treatment modalities.^[6–8]^ Furthermore, no studies have shown the incidence of distal ulnar fracture in distal ulnar fractures among adults of different ages in China. Therefore, this retrospective study aimed to investigate the incidence of distal radial fractures with and without associated distal ulnar fractures among adults in southern China.

## 2. Patients and methods

This retrospective study collected the following data: patient demographics (sex and age), mechanism of injury, and time of injury. Using the hospital’s Intranet (Uniform Resource Locator: 10.36.255.213, Yi Lai DA Digital Medical Imaging System), all data were accessed and processed from January 1, 2022, to December 31, 2022. Between January 2010 and December 2020, 2333 patients presented to the authors’ institution with distal radius fractures. Details of all patients presenting with X-ray-confirmed distal radius fractures could be used to investigate the incidence of distal radius fractures with or without distal ulnar fracture, including patients aged 18 years and over in a southern China county.

**Figure 1.**
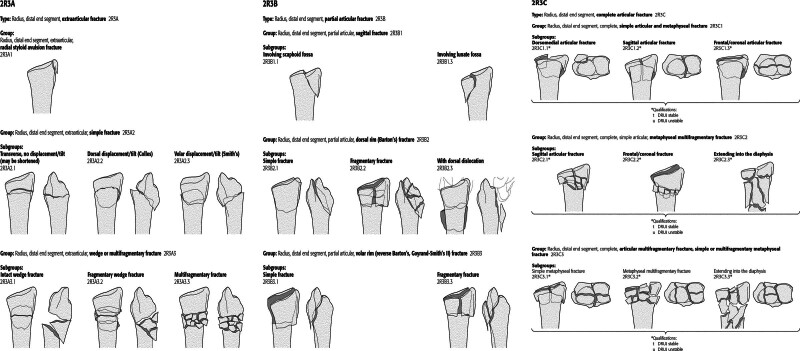
Classification of distal radius fractures is classified according to AO/OTA classification (https://classification.aoeducation.org/files/download/AOOTA_Classification_2018_Compendium.pdf).

We conducted a retrospective study to investigate the incidence of distal radial fractures with and without associated distal ulnar fractures among adults in southern China. All patients had acute fractures, and 2333 patients (2351 wrists) met the inclusion criteria. The inclusion criteria were as follows: (1) patients with a distal radius fracture from January 1, 2010, to December 31, 2020; (2) aged 18 years or older; and (3) residing in the county of **, China, at the time of injury. Two independent orthopedists classified the fractures as types A, B, or C according to the AO classification (Fig. 1).

Using ICD-10 codes S52.5 and S52.6, we searched the Hospital Information System for all patients who had been diagnosed with a distal radius fracture between January 1, 2010, and December 31, 2020. Patients diagnosed at ** County People’s Hospital but living outside ** County were excluded, and all data were registered retrospectively in a database.

This study was approved by the Ethics Committee of ** Hospital (No. 2021010101). The clinical and radiographic data of patients with distal radial fractures were reviewed and analyzed in this study. The treatment process did not change and was not harmful to the patient, and the ethics committee supervised the entire process of the experiment and strictly ensured the privacy of the patients.

**Figure 2.**
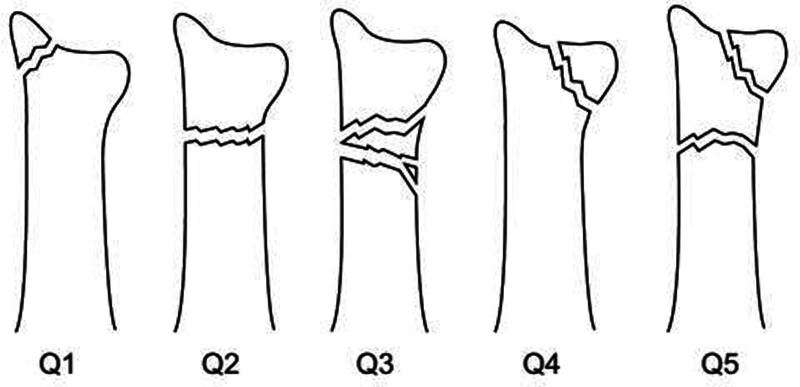
Classification of distal ulna fractures is classified using a Q system (Kim et al, 2016).

Disagreements were addressed through a discussion of 5 different Q-types according to the distal ulnar fracture location (Fig. 2). Fractures of the ulnar styloid were divided into either base or tip fractures, depending on whether the fracture level was proximal to the ulnar head on anteroposterior radiographs. Q1 was subclassified into tip and base types of styloid fractures. Disagreements were resolved by discussion and consensus.^[1]^

The peak incidence of distal radius fractures occurred in two age groups: children younger than 15 years and adults over 50 years.^[9]^ Consequently, patients were stratified into 2 distinct groups based on age: 18 to 49 years old and 50 or older. Within these groups, patients were further categorized into specific age ranges: 18 to 22, 23 to 27, 28 to 32, 33 to 37, 38 to 42, 43 to 47, 48 to 52, 53 to 57, 58 to 62, 63 to 67, 68 to 72, 73 to 77, 78 to 82, 83 to 87, 88 to 92, 93 to 97, and ≥98 years.

### 2.1. Statistical analysis

IBM SPSS Statistics (Version 23.0) was used to perform the statistical analysis on a personal computer. Descriptive statistical data were reported as means ± standard deviation (x ± SD) or percentages (%). The Mann–Whitney *U* test was used for continuous variables, and the chi-square or Fisher exact test was used for categorical variables. The significance level was set at *P* < .05.

## 3. Results

Between January 1, 2010, and December 31, 2020, 2333 patients (2351 fractures) with a mean age of 58.79 ± 13.79 years (range: 18–103) satisfied the inclusion criteria. In total, 1719 females (73.68%) and 614 males (26.32%) were included in this study. The female-to-male ratio of distal radial fractures was approximately 2.8 to 1 (or 74:26). Only 0.72% of all distal radial fractures (n = 17/2351) were open distal radius fractures, while 0.81% of distal radial fractures were accompanied by older distal ulnar fractures (n = 19/2351). The incidence of fresh distal ulnar fractures was 49.81% (n = 1171/2351 sides) (Table 1).

**Table 1:** Proportion of distal ulna fractures in adults’ distal radius fractures in the region of **, China, during 2010 to 2020.

		Female		Male	
		18–49 (year)	≥50 (year)	18–49 (year)	≥50 (year)
Mean age		42.09 ± 6.83	64.44 ± 10.11	38.64 ± 8.74	61.86 ± 9.75
Open distal radius fractures (n = 17, 0.72%)		1	7	6	3
With old distal ulna fractures (n = 19, 0.81%)		0	14	1	4
Without distal ulna fractures (n = 1161, 49.38%)		134, 63.51%	639, 42.09%	198, 65.35%	190, 59.56%
With fresh distal ulna fractures (n = 1171, 49.81%)		77, 36.49%	865, 56.98%	104, 34.32%	125, 57.07%
Distal radius fracture	Total (n = 2333)	210	1509	297	317
	Unilateral (n = 2315)	209, 99.5%	1500, 99.4%	291, 97.9%	315, 99.4%
	Bilateral (n = 18)	1, 0.5%	9, 0.6%	6, 2.1%	2, 0.6%
Injured extremity	Total (n = 2351)	211	1518	303	319
	Right (n = 1129)	104, 49.29%	735, 48.42%	133, 43.89%	157, 49.22%
	Left (n = 1222)	107, 50.71%	783, 51.58%	170, 56.11%	162, 50.78%
AO type of distal radius fracture	Total (n = 2351)	211	1518	303	319
	A (n = 671, 28.54%)	44, 20.9%	440, 29.0%	85, 28.1%	102, 32.0%
	B (n = 754, 32.07%)	93, 44.1%	369, 24.3%	167, 55.1%	125, 39.2%
	C (n = 926, 39.38%)	74, 35.1%	709, 46.7%	51, 16.8%	92, 28.8%

The study included 1161 patients (49.38%) with isolated distal radial fractures and 1092 patients (46.44%) with associated fractures of the ulnar styloid. In females, the rate of distal radial fractures accompanying distal ulna fractures was 36.49% (range: 18–49 years, n = 77/211) and 56.98% (range: 50 or older, n = 865/1518). In males, the rate of distal radial fractures accompanying distal ulna fractures was 34.32% (range: 18–49 years, n = 104/303) and 57.07% (range: 50 or older, n = 125/219). There were no significant differences between females and males within the same age group (*P* = .000).

**Figure 3.**
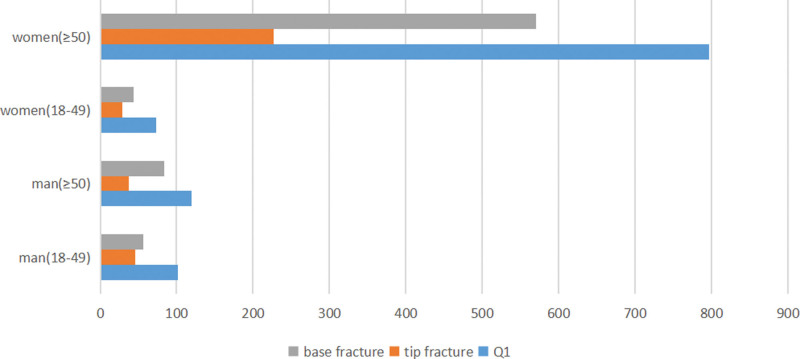
The number of patients with ulna styloid fractures according to tip or base fracture in Q1 type ulna styloid fractures.

Of all patients, 0.7% (n = 18/2333) had bilateral distal radial fractures and only one was open in these cases. In females, the distal radial fractures were unilateral (range: 18–49 years, n = 209, 99.5%; range: 50 or older, n = 1500, 99.4%). In males, the distal radial fractures were also unilateral (range: 18–49 years, n = 291, 97.9%; range: 50 or older, n = 315, 99.4%). There were significant differences between females and males (*P* = .000) (Table 1, Fig. 3).

Distal radius fractures classified as AO type C (926, 39.38%) constituted the majority of the fractures, followed by type B (754, 32.07%) and type A (671, 28.54%) fractures in adults. Among women, we identified 484 (27.99%) type A fractures, 462 (26.72%) type B fractures, and 783 (45.29%) type C fractures. Among men, we identified 187 (30.06%) type A fractures, 292 (46.95%) type B fractures, and 143 (22.99%) type C fractures. The chi-square test revealed significant differences between females and males (*P* = .000).

**Figure 4.**
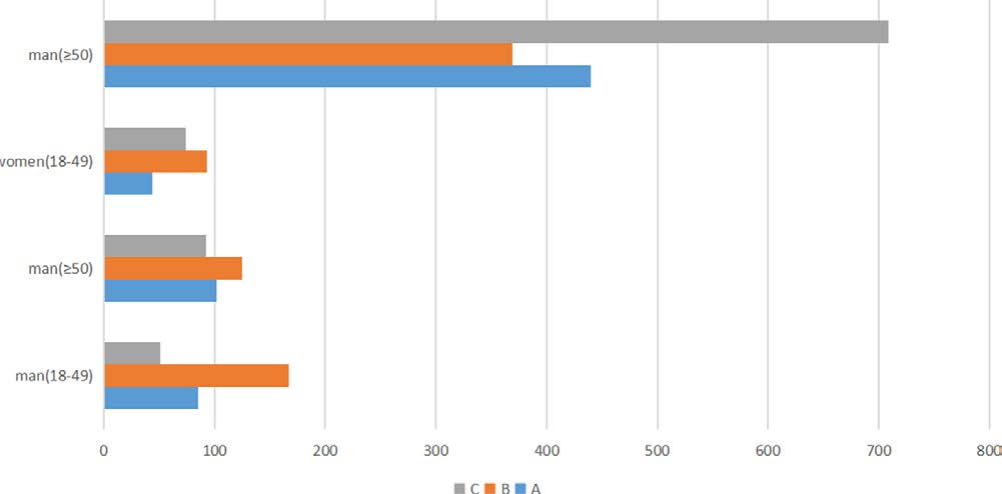
The number of patients with distal radius fractures at different age groups according to the AO/OTA fracture classification.

According to the AO/OTA fracture classification, distal radius fractures in females aged 18 to 49 years were distributed as follows: 44 (A, 20.9%), 93 (B, 44.1%), and 74 (C, 35.1%). These distributions were as follows in females aged 50 years or older: 440 (A, 29.0%), 369 (B, 24.3%), and 709 (C, 46.7%). In males aged 18 to 49 years, the distributions were: 85 (A, 28.1%), 167 (B, 55.1%), and 51 (C, 16.8%). In contrast, these were 102 (A, 32.0%), 125 (B, 39.2%), and 92 (C, 28.8%) in males aged 50 or older. There were significant differences between the women and men (*P* = .000) (Table 1, Fig. 4). We found that more females (range: 50 or older) had type C distal radius fractures compared to males; however, ocular type B fractures occurred more frequently in males than in females (range: 18–49 years).

The mean age of female patients was 61.71 ± 12.13 years, while for male patients, it was 50.63 ± 14.86 years, indicating significant differences between females and males (*P* = .000). Further stratification by age revealed that the mean age for females was 42.09 ± 6.83 (18–49 years) and 64.44 ± 10.01 (50 or older). Similarly, for males, the mean age was 38.64 ± 8.74 (18–49 years) and 61.86 ± 9.75 (50 or older years), with no significant differences between females and males within the same age group (*P* = .000).

**Figure 5.**
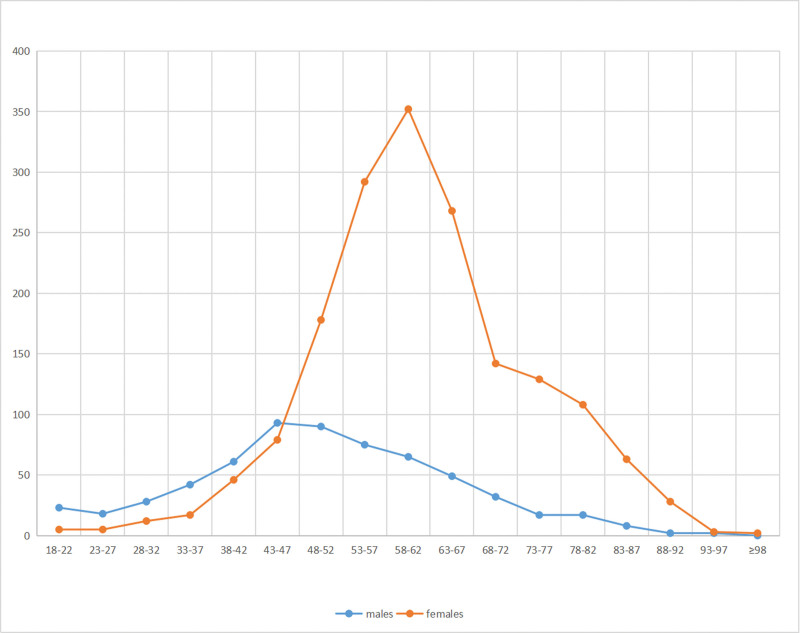
The number of distal radius fractures in adults in the region of Suichang, China, during 2010 to 2020 according to age group and sex.

**Figure 6.**
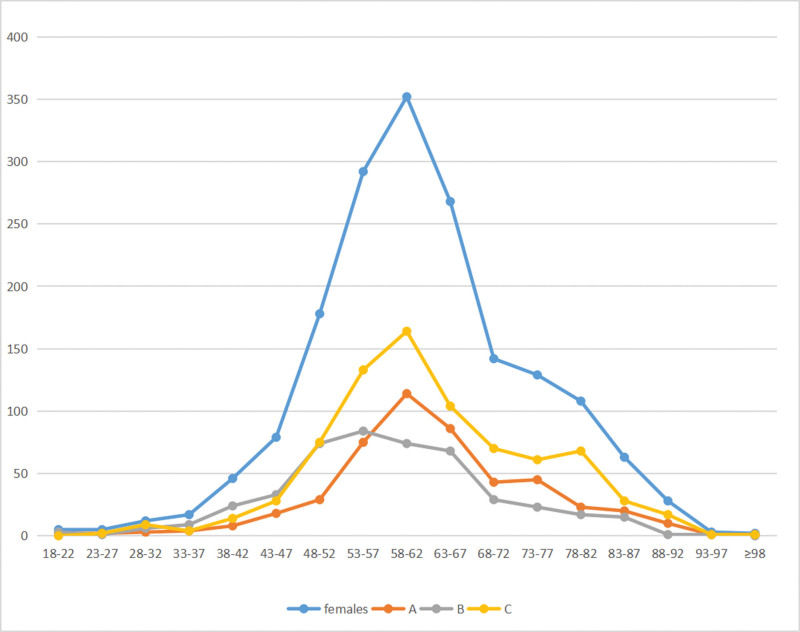
The number of distal radius fractures at different age groups in females.

**Figure 7.**
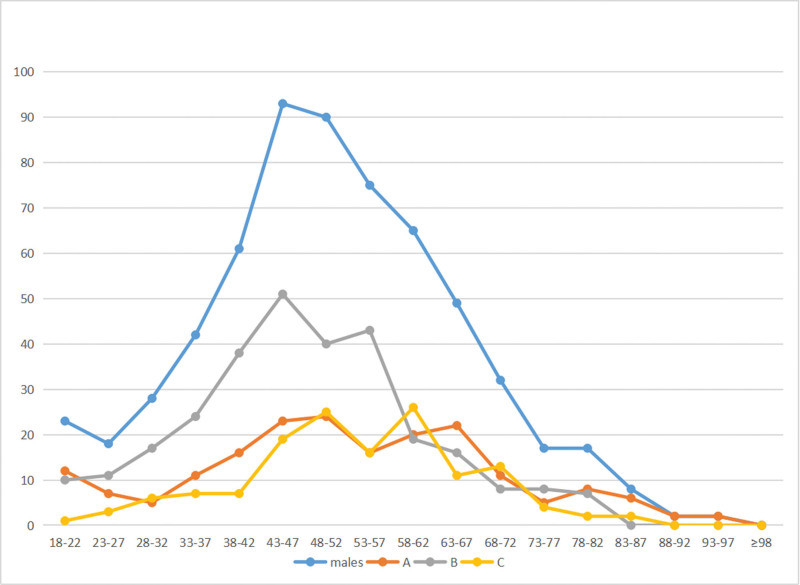
The number of distal radius fractures at different age groups in males.

To calculate the incidence of different fracture types in relation to age, the study grouped patient radiographs into 17 categories based on age ranges. The patients were categorized as follows (Figs. 5, 6, and 7).

The peak incidence of distal radius fractures was observed in males aged 43 to 47 years. In contrast, among women, an exponential increase in incidence occurred between the ages of 58 to 62. Specifically, type A distal radial fractures peaked in females between 53 and 57 years, while type B and C fractures reached their highest incidence in females aged 58 to 62 years. For males, the peak incidence of type B distal radius fractures occurred from 43 to 47 years, exhibiting a bimodal distribution with peaks in both younger and elderly males. Overall, the peak incidence of distal radius fractures in males was markedly smaller than in females in different age groups.

There were 6 different Q classifications, with Q1 referring to styloid fractures. The most common distal ulnar fracture type was Q1 of the ulnar styloid (1093, 93.25%), followed by Q5 fractures (39, 3.33%), while Q4 was the least common (11, 0.93%). Nevertheless, both Q2 (15, 1.28%) and Q3 (13, 1.11%) also exhibited relatively lower occurrences. Moreover, Q2 and Q4 fracture types were not observed in males (Table 2).

**Table 2:** Distribution of distal ulna fractures in adults according to Q-type, in the region of **, China, during 2010 to 2020.

Q-type (n = 1171)	Females	Males
Q1 (n = 1093, 93.25%)	870, 79.6%	223, 20.4%
Q2 (n = 15, 1.28%)	15, 100%	0
Q3 (n = 13, 1.11%)	8, 61.54%	5, 38.46%
Q4 (n = 11, 0.93%)	11, 100%	0
Q5 (n = 39, 3.33%)	38, 97.44%	1, 2.56%
Total	942, 80.44%	229, 19.56%

Ulnar styloidal base fractures were more common at 69.02% (n = 754/338 sides) compared to tip fractures at 30.98%. The ratio between base and tip fractures was approximately 2.23 to 1. Q1 distal ulnar fractures were distributed as follows in females aged 18 to 49 years: 73 (Q1, 34.59%), 29 (tip, 13.74%), and 44 (base, 20.85%). These were distributed as follows in females aged 50 or older: 796 (Q1, 52.44%), 226 (tip, 13.89%), and 570 (base, 37.55%). Meanwhile, the distributions in males aged 18 to 49 years were 102 (Q1, 33.66%), 46 (tip, 15.18%), and 56 (base, 18.48%). For males in the range of 50 or older years, the distribution was 121 (Q1, 37.93%), 37 (tip, 11.60%), and 84 (base, 26.33%) (Table 3).

**Table 3:** Distribution of distal ulna fractures in adults according to Q1-type, in the Region of **, China, during 2010 to 2020.

		Female (n, %)		Male (n, %)	
		18–49 (year)	≥50 (year)	18–49 (year)	≥50 (year)
Q1 fracture	Total (n = 1092)	73 (34.59%)	796 (52.44%)	102 (33.66%)	121 (37.93%)
	Tip (n = 338)	29 (13.74%)	226 (13.89%)	46 (15.18%)	37 (11.60%)
	Base (n = 754)	44 (20.85%)	570 (37.55%)	56 (18.48%)	8426.33%

## 4. Discussion

This retrospective study aimed to determine whether distal ulnar fractures are related to distal radial fractures. Our review shows that 49.81% of fractures of the distal radius were associated with fractures of the distal ulna. Additionally, 46.44% of fractures of the distal radius were associated with fractures of the ulnar styloid, a ratio that is similar to a review in which about 44% to 65% of fractures of the distal radius were accompanied by ulnar styloid fractures.^[3,9–11]^ During the study period, we found that the most common distal ulnar fracture type was the ulnar styloid Q1 type (93.25%). This result seemingly contradicts a study conducted in Östergötland, Sweden, between 2010 and 2012, which was published in 2020, indicating that the most common fracture type was the ulnar styloid Q1 type.^[3]^ We posit that this discrepancy may stem from the likelihood that our study enrolled a larger number of patients than the latter due to the extended follow-up period.

Only 0.72% of all distal radial fractures were open, which is similar to the result of a Stockholm area study in which the proportion was 0.8%,^[12]^ while it is less than that reported in a Swedish national study (1.2%).^[13]^ Meanwhile, 0.7% had bilateral distal radial fractures in our study. In men, the incidence of bilateral fractures of the distal radius was significantly more than women. This finding is in accordance with some previous study^[14–16]^ which bilateral distal radius fracture is rare, with few case series in the literature, even in large trauma care centers, and the higher prevalence of bilateral fractures in men, for example, who usually experience high-energy trauma.^[17,18]^

In the examination of 27,615 cases, Viberg et al identified a 31% increase in distal radial fractures over a 22-year period. This surge was primarily attributed to the growing elderly population, with a particularly notable rise among women and individuals aged 50 to 69 years.^[11]^ Meanwhile, Wilcke et al studied 42,583 patients with distal radial fractures and evaluated their age, sex, and surgical treatments.^[12]^ They found that the incidence rate in women increased rapidly, leveling off at a high age after 45 years, while men exhibited a lower incidence that increased gradually until the age of 80 years. Surprisingly, our research showed that the peak incidence of distal radius fractures occurred in males aged 43 to 47 years. Conversely, among women, we found an exponential increase in incidence between the ages of 58 and 62 years. This observed difference may be attributed to the fact that men and women were counted separately in our study. In a different study, when examining 1279 cases of unilateral distal radial fractures, Herzberg and Castel^[4]^ found that 61% of patients were female, with a mean age at the time of injury recorded at 56 years (16–102 years). These patients were classified as type A (383 cases, 30%), type C (809 cases, 63%), or type B (87 cases, 7%), and notably, no distal ulnar fractures were observed in type B cases. These results were in accordance with our findings which distal radius fractures classified as AO type C (926, 39.38%) constituted the majority of the fractures, followed by type B (754, 32.07%) and type A (671, 28.54%) fractures in adults. Meanwhile, another study by Rundgren et al^[13]^ found that the majority (65%) of all distal radius fractures were extra-articular (AO-23-A), 12% were partially articular (AO-23-B), and 23% were complete intra-articular (AO-23-C).

The female-to-male ratio of distal radial fractures was approximately 2.8 to 1 (74:26) in our study. Menopause is a natural phenomenon among middle-aged women and most go through it around 50 years old.^[19]^ The obvious increase in the number of fractures in postmenopausal women can explain why more than 70% distal radial fractures occurred in women aged over 50 years. The ratio between women and men of 74:26 found in this study is similar to the result of a Swedish regional study,^[20]^ while it is slightly lower than that reported in two Swedish national study; 78:22^[13,21]^ and considerably higher than what a British regional study reported; 68:38.^[22]^

Studies have shown that distal radius fractures are an overall higher incidence in middle-aged and elderly women, and patients with distal radius fractures have a high incidence of osteoporosis.^[23–26]^ In our study, the results showed that the incidence of distal radius fractures in males was notably smaller than that in females across different age groups. Specifically, more females (range: 50 years or older) had type C distal radius fractures than males. Conversely, type B fractures were more frequently documented in males within the 18 to 49 age range. In our perspective, osteoporosis stands out as the primary cause of fractures in women aged over 50 years, especially considering that several studies have indicated that low-energy distal radial fractures are the most common upper-arm fractures associated with bone fragility.^[27–29]^ However, this results appear to contradict a research (in this observational study of 289 consecutive patients aged ≥40 years with a distal radius fracture were included) published earlier several years ago showing that patients with osteoporosis did not have increased odds of a more complex distal radius fracture (type B + C, n = 192; type A, n = 92) compared to those with osteopenia/normal BMD (n = 159) and patients with AO fracture types A or C had a higher prevalence of osteoporosis than patients with type B fracture.^[30]^ We hold the opinion that more young people were included in our research is the main reason which results in such result.

The present study has some limitations. First, it included only retrospective cases of distal radial fractures treated at a general county-level hospital. Therefore, the incidence of radius fractures in adults of different ages in China cannot be reliably determined. Second, reliance on X-ray evaluations for distal radius and ulnar fractures introduces the potential for misdiagnosis and missed diagnoses, despite the clinician’s expertise. The utilization of computed tomography (CT) could enhance diagnostic accuracy and reduce the risk of misdiagnosing fractures. Third, radiography has been proven to be less sensitive than CT in diagnosing ulnar styloid fractures. However, routine CT scans are considered impractical owing to their high medical costs. Additionally, some patients exhibited congenitally small or variable ulnar styloid processes, potentially impacting the accurate diagnosis and classification of ulnar styloid fractures.

In conclusion, about half of distal radius fractures were associated with distal ulnar fractures, and the Q1 type (encompassing 3 fracture types: tip, neck, and base) was the most common type of distal ulnar fracture. We found that more females (age range: 50 years or older) had type C distal radius fractures than males. However, ocular type B fractures occurred more frequently in males than in females (range: 18–49 years). From our standpoint, osteoporosis emerges as the predominant factor contributing to fractures in women aged over 50 years. Furthermore, the highest occurrence of radius fractures in males was consistently lower than that observed in females across various age groups.

## Author contributions

**Conceptualization:** Wu Zeng, Jun-Feng Zhu, Jun Li.

**Data curation:** Zhe-Kang Huang, Jun Li.

**Investigation:** Jun-Feng Zhu.

**Methodology:** Jun-Feng Zhu, Jun Li.

**Project administration:** Jun Li.

**Software:** Zhe-Kang Huang.

**Supervision:** Zhe-Kang Huang, Jun-Feng Zhu.

**Validation:** Jun-Feng Zhu.

**Visualization:** Jun-Feng Zhu.

**Writing – original draft:** Wu Zeng, Zhe-Kang Huang.

**Writing – review & editing:** Wu Zeng, Zhe-Kang Huang.
